# 1例复合性小细胞肺癌病例及文献复习

**DOI:** 10.3779/j.issn.1009-3419.2025.101.15

**Published:** 2025-09-20

**Authors:** Minglang GAO, Xiao LU, Bo HAO, Ning LI, Songping XIE

**Affiliations:** 430060 武汉，武汉大学人民医院胸外科; Department of Thoracic Surgery, Renmin Hospital of Wuhan University, Wuhan 430060, China

**Keywords:** 肺肿瘤, 复合性小细胞肺癌, 鳞癌, 病例报告, 文献复习, Lung neoplasms, Combined small cell lung cancer, Squamous cell carcinoma, Case report, Literature review

## Abstract

复合性小细胞肺癌（combined small cell lung cancer, CSCLC）是根据世界卫生组织2015年肺癌新病理分类，将SCLC与非小细胞肺癌（non-small cell lung cancer, NSCLC）成分混合而成的癌症，在神经内分泌肿瘤中被归类为SCLC的亚型。目前对于SCLC的研究主要集中于单一成分的纯SCLC，对于CSCLC的研究相对较少，且CSCLC临床罕见，尚无规范的治疗方案，对于CSCLC的临床病理特征及预后预测指标缺乏统一的认知，需要进一步观察疗效和预后。现报告1例CSCLC患者的诊疗经过，并对CSCLC的研究现状进行文献回顾。

## Abstract (en)

Combined small cell lung cancer (CSCLC) is a cancer that mixes small cell lung cancer (SCLC) with non-small cell lung cancer (NSCLC) components according to the World Health Organization's 2015 New Pathologic Classification of Lung Cancer. Composed of a mixture of SCLC and NSCLC components, CSCLC is classified as a subtype of SCLC in neuroendocrine tumors. Currently, research on SCLC mainly focuses on single-component pure SCLC, with relatively few studies on CSCLC, which is clinically rare and has no standardized treatment protocols and lacks a unified perception of the clinicopathological features and prognostic predictive indexes of CSCLC. Further observation of efficacy and prognosis is needed. We report the treatment course of a case of CSCLC and provide a literature review of the current status of research on CSCLC.

肺癌死亡率高居全球肿瘤相关死亡率榜首，其病理类型主要包括非小细胞肺癌（non-small cell lung cancer, NSCLC）和小细胞肺癌（small cell lung cancer, SCLC）^[1]^。在我国，肺癌以NSCLC为主，并以其高转移性为主要特征^[2]^，而SCLC具有高侵袭性、早期转移和预后不良的特点^[3]^。随着人们对肺癌研究的深入了解，发现了一种混合了SCLC和NSCLC成分的病理类型肺癌，即复合性小细胞肺癌（combined SCLC, CSCLC）。CSCLC最初于1981年由世界卫生组织首次确定为SCLC的一个亚型，并于1999年正式分为单纯SCLC和CSCLC。CSCLC中的NSCLC成分可以是鳞状细胞癌（lung squamous cell carcinoma, LUSC）、腺癌（lung adenocarcinoma, LUAD）、大细胞神经内分泌肿瘤（large cell neuroendocrine carcinoma, LCNEC）等，混合的成分可以是一种，也可以是多种，目前最常见的是混合LUSC^[4]^。然而，更新版的《NCCN小细胞肺癌临床实践指南》重新定义了CSCLC，将含有至少10%的LCNEC的SCLC混合成分的肿瘤形式也包括在内，可被归类为CSCLC^[5]^。这一重新定义反映了SCLC和LCNEC之间的组织学连续性，使得CSCLC的临床区分更具有挑战性。

目前，绝大多数肺癌研究聚焦于单纯SCLC或NSCLC，对CSCLC的系统性研究较为有限。由于其发病率低、组织学构成复杂，临床诊断常依赖术后病理，治疗上亦缺乏统一规范。随着精准医疗与多学科诊疗模式的发展，深入探讨CSCLC的临床病理特征、分子机制及治疗反应，对于改善患者预后具有重要意义。本文报告1例CSCLC患者的诊疗过程，并结合相关文献进行回顾，旨在为临床工作者提供参考，推动对该疾病更深入的认识与研究。

## 1 病例资料

患者男，71岁， 2023年10月6日因咳嗽一月余行胸部CT检查发现左下肺占位性病变，肺癌可能性大（图1）。当地医院对此肿块进行射频消融术治疗，术后即出现气胸、皮下气肿，予以对症治疗十天未见明显改善，转入武汉大学人民医院就诊。患者既往有冠状动脉粥样硬化性心脏病病史10年，慢性阻塞性肺疾病病史5年，心脏支架置入术后7年，吸烟史45年（每天半包，未戒烟）。初步诊断：气胸；皮下气肿；左下肺占位性改变，肺癌可能；慢性阻塞性肺病。

**图1:**
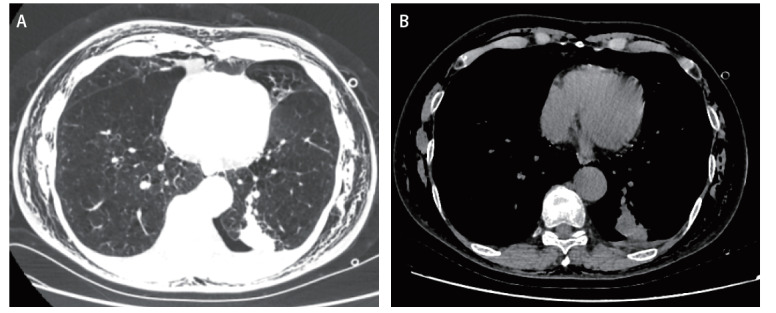
胸部CT平扫提示左肺占位性病变。A：手术前胸部CT检查肺窗；B：手术前胸部CT检查纵隔窗。

入院后完善相关术前检查，考虑患者存在肺功能差，长时间肺部漏气不能愈合，且肺部肿块可以局部切除。充分沟通后于2023年10月9日全身麻醉下行胸腔镜下肺楔形切除术+胸膜粘连松解术+淋巴结清扫术，术中探查见：左肺与胸壁膜状粘连，全肺呈气肿状，顺应性差，左下肺肿块，直径大小约2.5 cm，质硬；并此处肺组织存在漏气。术后第7天愈合出院。

病理检查结果：（1）（左下肺肿块）CSCLC，SCLC成分约60%+LUSC成分约40%（中间分化），肿瘤直径2 cm，未见明确的癌栓及脉管内神经侵犯，未见脏层胸膜侵犯，未见气腔播散。（2）吻合口的切缘组织呈慢性炎症，未见癌。（3）免疫组化结果显示：SCLC：CD56（+）、甲状腺转录因子-1（thyroid transcription factor-1, TTF-1）（+）、突触素（Synaptophysin, Syn）（+）、CgA（+）、CK7（-）、 Ki-67（+，约80%）；LUSC：CK5/6（+）、CK7（-）、Ki-67（+，约30%）、NapsinA（-）、P40（+）、P63（+）、TTF-1（-）（图2）。第5组、第7组、第8组、第10组见淋巴结0/1、0/1、0/1、 0/1枚，未见癌转移。

**图2:**
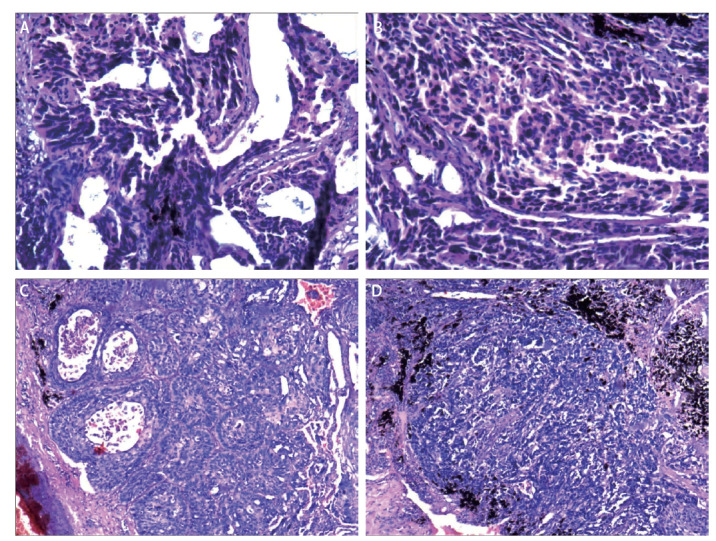
冰冻病理切片镜下图。A：以SCLC为主（细胞小、胞质少、核深染），局部散在LUSC成分（多边形细胞，胞质略丰富）（HE染色，×400）；B：SCLC密集分布，边缘区域可见少量LUSC细胞（呈团状，有细胞间桥倾向）（HE染色，×400）；C：SCLC与LUSC混合分布（LUSC呈小团状，胞质丰富）（HE染色，×200）；D：LUSC成分明显（细胞呈铺路石样排列，有细胞间桥），伴SCLC区域（HE染色，×200）。

患者出院后回当地医院肿瘤科予以辅助化疗（卡铂+紫杉醇），6个月后来我院进行复查头颅磁共振成像（magnetic resonance imaging, MRI）、胸腹部计算机断层扫描（computed tomography, CT）和肿瘤标记物等，未发现转移征象，结果如图3和表1所示。图3手术前后胸部CT平扫检查对比。

**图3:**
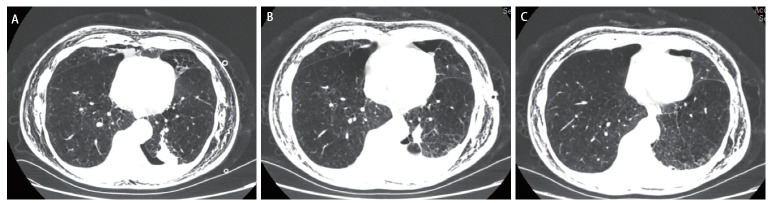
手术前后胸部CT平扫检查对比。A：手术前1周胸部CT检查；B：手术后两周胸部CT复查；C：手术后6个月（已完成4个周期化疗）胸部CT复查。

**表1:** 治疗前后血清肿瘤标志物比较

Time	Carcinoembryonic antigen (μg/L)	Carbohydrate antigen 125 (U/mL)	Cytokeratin-19 fragment (μg/L)	Neuron-specific enolase (μg/L)
Preoperative	5.21↑	13.00	3.42↑	21.40↑
2 weeks after surgery	2.13	11.00	2.90	18.00↑
6 months after surgery	2.07	10.80	2.97	16.80↑
Reference range	0.00-5.00	0.00-24.00	0.00-3.30	0.00-16.30

该病例报道已取得患者知情同意。

## 2 讨论

CSCLC的起源仍不明确，可能是患者体内两种或以上不同病理类型的肺癌发生了融合^[6]^，也可能是不同病理类型之间的相互转换，例如患者在使用表皮生长因子受体-酪氨酸激酶抑制剂（epidermal growth factor receptor-tyrosine kinase inhibitors, EGFR-TKIs）治疗后会产生耐药作用^[7,8]^，即可能发生了NSCLC向SCLC的转换^[9]^。在临床特征方面，CSCLC和SCLC在年龄和肺转移方面没有差异。然而，在种族、性别、T分期、N分期、手术、骨转移、脑转移和肝转移等方面观察到显著差异。研究^[4,10]^表明，早期CSCLC（I和II期）的发病率远远高于单纯SCLC，29%的CSCLC患者被诊断为I-II期，而SCLC患者只有10%。另外，CSCLC患者大多数具有常年吸烟史，与SCLC主要致病因素类似^[11,12]^。CSCLC的诊断以病理检查为主，但手术的取材范围和标本大小有限，如术前淋巴结穿刺活检、计算机断层扫描（computed tomography, CT）引导下肺穿刺活检、纤维支气管镜下肺活检等，很难反映肿瘤的真实病理类型特征，且诊断率较低^[13]^。有研究^[14]^发现170例SCLC中CSCLC发生率仅为5.9%，其中包括5例LUSC，3例LCNEC和2例LUAD，可见CSCLC发生率很低。在病理检查中的免疫组化（immunohistochemistry, ICH）方面，主要肿瘤标志物有CD56、Syn、CgA、TTF-1、CK、Ki-67等，其中CD56、Syn、CgA属于神经内分泌肿瘤的标志物。

据文献^[15]^报道，CSCLC在SCLC中的发生率为1%-2%，无明显的特异性临床表现及影像学表现，确诊主要依靠病理学检查。目前针对CSCLC主要采取以化疗和放疗为主的综合治疗^[16]^，但其预后相比单纯SCLC较差^[17]^。根据2023版中国临床肿瘤学会（Chinese Society of Clinical Oncology, CSCO）《小细胞肺癌诊疗指南》^[18]^，对于局限期和广泛期的CSCLC患者，其治疗方案可参照单纯SCLC，即对于局限期的患者把手术治疗合并辅助放、化疗作为主要治疗方案，对于广泛期的患者行化疗合并免疫治疗，如果患者出现脊髓压迫症、上腔静脉综合征、骨转移或脑转移等局部症状，则行补充化疗及放疗。而对于合并非鳞非SCLC成分的CSCLC患者，指南建议进行基因检测，伴有驱动基因突变者可考虑联合靶向治疗；合并LUSC成分的CSCLC可联合免疫治疗。驱动基因突变在SCLC中非常少见，据报道*EGFR*基因突变在单纯SCLC中发生率约为4%，而在CSCLC中可达到15%-20%，多发生在无或有轻度吸烟史且混合LUAD成分的CSCLC。由于临床上并未对SCLC进行常规基因检测，实际突变率可能更高。目前已经报道多例携带*EGFR*突变的SCLC和混杂LUAD成分的CSCLC接受EGFR-TKIs治疗有效的个案，这提示靶向治疗可能在混杂LUAD成分且合并驱动基因突变CSCLC的治疗中具有潜在获益。

SCLC多存在基因组和染色体高度不稳定性，造成突变频率增加，理论上可能对免疫治疗更敏感。据报道CSCLC中同样存在*TP53*、*RB1*、*PTEN*等大量高频突变^[19]^，*RUNXIT1*扩增、YAPI表达具有一定特异性^[20]^。免疫治疗已在SCLC治疗中取得突破，然而免疫治疗对于CSCLC仍是未来需要深入探索的全新领域。病例报道^[21]^显示合并LUSC成分的CSCLC对免疫治疗有一定疗效。对于本例患者，其在接受手术治疗和后续常规的化疗（卡铂+紫杉醇）后，肿瘤标志物水平得到明显改善，这对无手术禁忌证的CSCLC患者的治疗具有一定的参考价值。

作为当前肿瘤治疗的热点，靶向治疗在近几年的NSCLC治疗研究中取得了重大突破，但SCLC和CSCLC的涉及面并不广。如今越来越多的CSCLC患者在放化疗的基础上配合外科手术治疗^[22,23]^，但手术在CSCLC早期阶段的作用仍不确定。然而，手术治疗对早期CSCLC患者非常有益。Hage等^[24]^评估了26例术后CSCLC患者，发现手术给I期CSCLC患者带来了长期的无病生存，甚至达到治愈效果。Babakoohi等^[4]^比较了22例CSCLC患者和406例单纯SCLC患者，并观察到手术治疗在CSCLC组患者中具有更高的优先级。Men等^[25]^报道了来自一家机构的114例CSCLC患者的结果，其中70.2%接受了两种或两种以上的治疗方案，手术组的5年总生存率高于非手术组（分别为48.9%和36.6%）。

综上所述，随着医疗技术的进步，CSCLC的诊断正在稳步增加，由此产生了对这一患者群体进行更广泛研究和改进治疗方法的迫切需求。对于IA-IB期的患者，手术干预可能是至关重要的，而对于IIA-IIIA期的患者，手术、化疗和放疗的组合可以有效地改善预后。对于晚期患者，应考虑以化疗为基础的治疗。然而，目前还没有普遍推荐的治疗CSCLC的方案。根据现有证据，CSCLC的治疗应遵循传统的SCLC指南，并应采取多学科综合治疗方法。未来迫切需要基于临床标本和动物模型的综合研究，利用创新的组织学技术，并通过多中心临床研究探索新的治疗策略。这种协调一致的努力对于增进我们对CSCLC的理解以及为CSCLC患者推进更有效和量身定制的治疗干预是至关重要的。
